# The effect of interpersonal relationship and epidemic attention on negative emotion among medical students: the mediating role of social satisfaction

**DOI:** 10.1186/s12888-023-05113-z

**Published:** 2023-08-21

**Authors:** Xiaoxue Chen, Binxin Huang, Wei Lin

**Affiliations:** 1 School of Intelligent Medicine, Chengdu University of Traditional Chinese Medicine, Chengdu, China; 2 School of Basic Medicine, Chengdu University of Traditional Chinese Medicine, Chengdu, China; 3 Shanghai Key Laboratory of Psychotic Disorders, Shanghai Mental Health Center, School of Medicine, Shanghai Jiao Tong University, Shanghai, China

**Keywords:** Interpersonal relationship, Epidemic attention, Social satisfaction, Negative emotion, Medical students

## Abstract

**Background:**

Individuals are required to avoid close contact to reduce the probability of contracting the virus during the epidemics, which can lead to social isolation and exacerbate interpersonal relationship issues. Social satisfaction plays a crucial role in management in the period of pandemics and is strongly correlated with negative emotion. Medical students, as a special group of students, have a heavier burden of academic workload and greater pressure. They are also more likely to have access to epidemic information, which increases their susceptibility to negative emotions such as depression and anxiety. Therefore, it is necessary to investigate the impact and mechanisms among interpersonal, epidemic attention, social satisfaction, and negative emotions during the epidemics outbreak among medical students for improving the level of mental health in the post-epidemic era.

**Methods:**

A total of 1,451 university students were included in this study. Self-administered questionnaires, including the Comprehensive Interpersonal Relationship Diagnostic Scale, the Self-Rating Anxiety Scale, and the Self-Rating Depression Scale, were utilized to construct structural equations to examine the mediating effects of social satisfaction. The study employed a multi-stage whole-group sampling approach for university students.

**Results:**

Interpersonal relationships and epidemic attention positively predicted negative emotion levels. Interpersonal relationships negatively predicted social satisfaction, while epidemic attention positively predicted social satisfaction. Moreover, social satisfaction negatively predicted negative emotion levels. Notably, both interpersonal relationships and epidemic attention indirectly affected negative emotions through the mediating effect of social satisfaction.

**Conclusions:**

Social satisfaction plays a mediating role in the effect of interpersonal relationships and epidemic attention on negative emotion. In the post-epidemic era, it is crucial to enhance support from family, school and society to improve social satisfaction of medical students. Immediate identification of negative emotions is essential, and targeted strategies should be developed to address mental health issues among medical students.

## Introduction

### Negative emotional reactions have become increasingly common during the epidemics

The occurrence of sudden public health events increases the negative emotion of the masses, and depression and anxiety are common psychological reactions [1]. Studies found that after the incidents, most respondents have negative emotional reactions such as anxiety and depression [2]. An online survey of the general population in China showed that more than 30% of the population suffered from psychological distress such as anxiety, depression, cognitive changes, etc. [3]. A survey conducted in Hong Kong found that 19% had depression and 14% had anxiety of the segregated respondents included [4]. While in Europe, studies sampling from UK showed that 22.12% had depression and 21.63% had anxiety among its samples during the early stages of the pandemics [5]. Research carried out in Ireland found that there are 20% of participants who had Generalized Anxiety Disorder (GAD) and 22.8% experienced depression [6]. Moreover, there was a research showed that individuals who feel less emotionally secure, become less satisfying and show more emotional reactivity as they behave more consciously and positively [7].

### Medical students are more prone to experiencing depression and anxiety

Medical students, as a special group of students, bear a heavier burden of schoolwork and face greater pressure, making them more susceptible to experiencing negative emotions, such as depression and anxiety [8, 9]. Previous surveys found that the student population of medical schools has a higher rate of emotional disorders than non-medical schools during the SARS epidemic [1], while the detection rate of depression and anxiety symptoms in medical students was higher during the new coronary pneumonia epidemic [10]. What is more, study conducted in Jordan found that pharmacy students had a higher level of depression and anxiety during the epidemics quarantine period [11].Negative emotions such as depression and anxiety easily lead to sleep disturbances, mood disorders, and abnormal activities, which affect social relationships and quality of life. Medical students are the main future frontline workforces in the hospitals, so they take more important and urgent responsibilities to reduce their own negative emotion levels not only for themselves but also for the future development of the medical industry.

### Loosened interpersonal relationship among masses during the epidemics

During the epidemics, due to the strong contagious power of the virus, it was suggested that individuals should avoid close contact to reduce the probability of contracting the virus, which can easily lead to social isolation [12]. Some studies believe that social isolation mainly refer to loose interpersonal relationships that lack contact and communication [13, 14], which significantly affect the mental health of the individuals after the epidemic [15, 16]. Its impact, which some researches has manifested, could enhance the individual’s sense of fear of related events and over-solve the external world, triggering avoidance behavior and negative emotions [17]. Some surveys showed that more than half of the individuals believe that during the epidemics, social interpersonal relationships tended to be tense, interpersonal vigilance was increased, and trust was lacking [18].However, interpersonal relationship significantly affects individuals’ mental health levels. Poor interpersonal relationships play an important role in generating negative emotions such as depression and anxiety [19].

### Epidemic attention rose for psychological safety

Public health emergencies refer to situations characterized by uncertainty, instability and insecurity. During crisis events, individuals often seek to gain a sense of control and alleviate anxiety by obtaining, disseminating, evaluating and utilizing information related to epidemics from different channels [20]. Research has found that uncertainty can lead to stress. During epidemics, smartphones become the primary source of information. Individuals who isolated and heavily rely on their smartphones to gain information can experience a sense of control that helps alleviate stress [21]. A study has shown that the degree and core of public regarding the epidemic has a significantly impact on public panic and mental health [22].

Therefore, it is essential to investigate the influencing factors, particularly interpersonal relationships and epidemic attention, on depression and anxiety during the period of epidemics and understand their underlying mechanisms of interaction with negative emotions.

## Methods

### Sampling methods

A cross-sectional survey was used to investigate the influence of interpersonal relationships and epidemic attention on negative emotions among university students, using convenience sampling and simple random sampling. A total of 1,500 questionnaires were distributed, 1,451 valid questionnaires were returned and the effective response rate was 96.7%. Epidata 3.1 was used for double entry of data, and structural equation models were constructed and tested using AMOS 23.0 software. The bias-corrected non-parametric percentile confidence interval Bootstrap method was used to test the mediating effect of social satisfaction between interpersonal relationships, epidemic concern and depression and anxiety using 1000 replicate samples.

### Research tools

#### The self-administered questionnaire

The self-designed questionnaire contained demographic variables, epidemic concern and social satisfaction.

The epidemic attention parts divided into four dimensions: total concern about the epidemic [23], time to read and view epidemic information, number of epidemic information obtained, and the frequency of reading and watching epidemic information. Social satisfaction part s divided into four dimensions[24]: satisfaction with community security, satisfaction with school epidemic prevention and control measures, satisfaction with the convenience of living and satisfaction with current life status. The self-administered questionnaire on epidemic attention and the self-administered questionnaire on social satisfaction are both scored on a 4-point scale. The higher the score on the self-administered epidemic concern scale, the higher the concern about the epidemic information, with a Cronbach’s of alpha 0.6 and relatively good reliability. The higher the score on the self-administered scale of social satisfaction, the lower the social satisfaction, with a Cronbach’s alpha of 0.7 and good reliability.

#### The comprehensive interpersonal relationship diagnostic scale

The Comprehensive Interpersonal Relationship Diagnostic Scale developed by Zheng Rizhao et al. was selected [25]. The scale is mainly used to measure the degree of interpersonal distress and consists of 28 questions divided into 4 dimensions: talking to individuals, interpersonal communication, treating individuals well and heterosexual communication. The higher the total score and the higher the score on each dimension, the more severe the degree of interpersonal distress. The scale has internal consistency reliability of 0.89. In this study, the Cronbach’s alpha measured by the scale was 0.86, with good reliability.

#### The self-rating anxiety scale

The Self-Rating Anxiety Scale is used to assess the severity of anxiety and consists of 20 items on a 4-point scale with positive and negative scoring, with the items summing up to a total score. The crude score was calculated and converted to a standard score by multiplying by 1.25, where a standard score of ≥ 50 was used to detect anxiety. In this study, the Cronbach’s alpha measured by the scale was 0.675, with relatively good reliability [26].

#### The self-rating depression scale

The Self-Rating Depression Scale is used to assess the severity of depression and consists of 20 items on a 4-point scale with positive and negative scoring, with the items summing up to a total score. The crude score was calculated and converted to a standard score by multiplying by 1.25, where a standard score of ≥ 50 was used to detect depressed mood. In this study, the Cronbach’s alpha measured by the scale was 0.748, with good reliability [27].

### Hypothesis

According to the theory of interpersonal relationship promoting growth model, good interpersonal relationships promoted individuals to actively respond to trauma-related events, encourage individuals to think positively, rebuild cognition, reduce individual psychological stress levels, and overcome negative psychological experiences [14]. Higher satisfaction in interpersonal relationships, to a certain extent, signify having good social support [28], and social support buffer the emotional response triggered by stressful events correspondingly [29]. During the epidemics, individuals tend to be socially isolated, and it is difficult to maintain satisfactory interpersonal relationships. Good interpersonal relationships effectively alleviate negative emotions such as depression and anxiety that are generated in the environment of the epidemics, and bad, broken, and loose interpersonal relationships exacerbate negative emotions.

H1: Interpersonal relationships positively predict depression and anxiety levels.

Various risk events exposed in the public’s vision during the epidemic made individuals feel threats from the external environment and were more likely to trigger negative emotions such as anxiety, depression, and panic [30, 31]. In an environment where risk events such as emergent public health events occur frequently, the more serious the threat perceived by the masses, the more frequent and more intense information searching behaviors are likely to occur [30]. The Audience-centric theory state that audiences are actively looking for the information they want [32]. Negative information, compared with positive or neutral information, bring a larger amount of information, thereby attracting more attention from the crowd [33]. Ecosystem theory and interpretation level theory also put forward similar views [34, 35]. However, paying attention to the epidemic information for a long time is more likely to trigger negative emotions [36]. Due to the peculiarities of the epidemic environment, most information about the epidemic spread through the Internet. Internet information are mixed and difficult to distinguish between false and true, which might cause psychological stress to netizens and affect their mental health [37]. Excessive time are spent focusing on epidemic information interferes with normal life and rest, manifested by a lack of necessary recreational activities or exercise, and exacerbates symptoms of depression and anxiety. Studies show that the increased frequency and intensity of epidemic information bring a large number of stimuli, resulting in perceptual deactivation and a weakened ability to regulate and process emotions. When facing with a sudden epidemic, individuals tend to behave as a decrease in processing capabilities, leading to an increase and accumulation of negative emotions [38]. Therefore, this study assumes that the degree of attention to the epidemic negatively predicts the level of depression and anxiety.

H2: Epidemic attention negatively predicts depression and anxiety levels.

Satisfaction refers to a state of mind that describes a person’s subjective evaluation of the quality of a relationship[39]. Depression and anxiety are closely related to satisfaction. Research on depression and anxiety emotions and satisfaction, from job satisfaction [40] to physical satisfaction [41] to nurse-patient relationship satisfaction etc., these results show that satisfaction negatively affects the level of anxiety and depression, and the increase in satisfaction is beneficial to reduce negative emotion. Among them, there are many surveys on negative emotions and life satisfaction, and it is found that the generation of anxiety and depression is negatively correlated with life satisfaction [42, 43]. In addition, satisfaction is also affected in many ways. In terms of interpersonal relationships, life satisfaction, job satisfaction, and community satisfaction are equal to interpersonal problems and are negatively correlated [44]. At the level of information attention, the public’s expectations and attention are positively affecting official media satisfaction[45].

Social satisfaction is a kind of satisfaction, and it is an important social psychological index, which refers to the perception and experience that individuals show to the social environment in work and life [46]. During the epidemics, due to the peculiarities of the environment, individuals’ interpersonal relationships, information attention, and negative emotion levels fluctuated, and social satisfaction also change. Social satisfaction plays a vital role in social harmony, social construction, and social development. However, there are few related studies on the influencing factors of social satisfaction and its relationship with negative emotions. Because of this, this study proposes the following hypotheses: social satisfaction negatively predicts depression and anxiety; interpersonal disturbance negatively affects social satisfaction; epidemic attention positively affects social satisfaction; social satisfaction is in interpersonal relationship disturbance and epidemic concern; the degree plays a partially mediating role in the influence of depression and anxiety levels.

H3: Social satisfaction negatively predicts depression and anxiety.

H4: Interpersonal disturbance negatively predicts social satisfaction.

H5: Epidemic attention positively predicts social satisfaction.

H6: Social satisfaction plays a partially mediating role in the influence of interpersonal relationships and epidemic attention on depression and anxiety levels.

## Results

### Description statistics and correlations

**Fig. 1:**
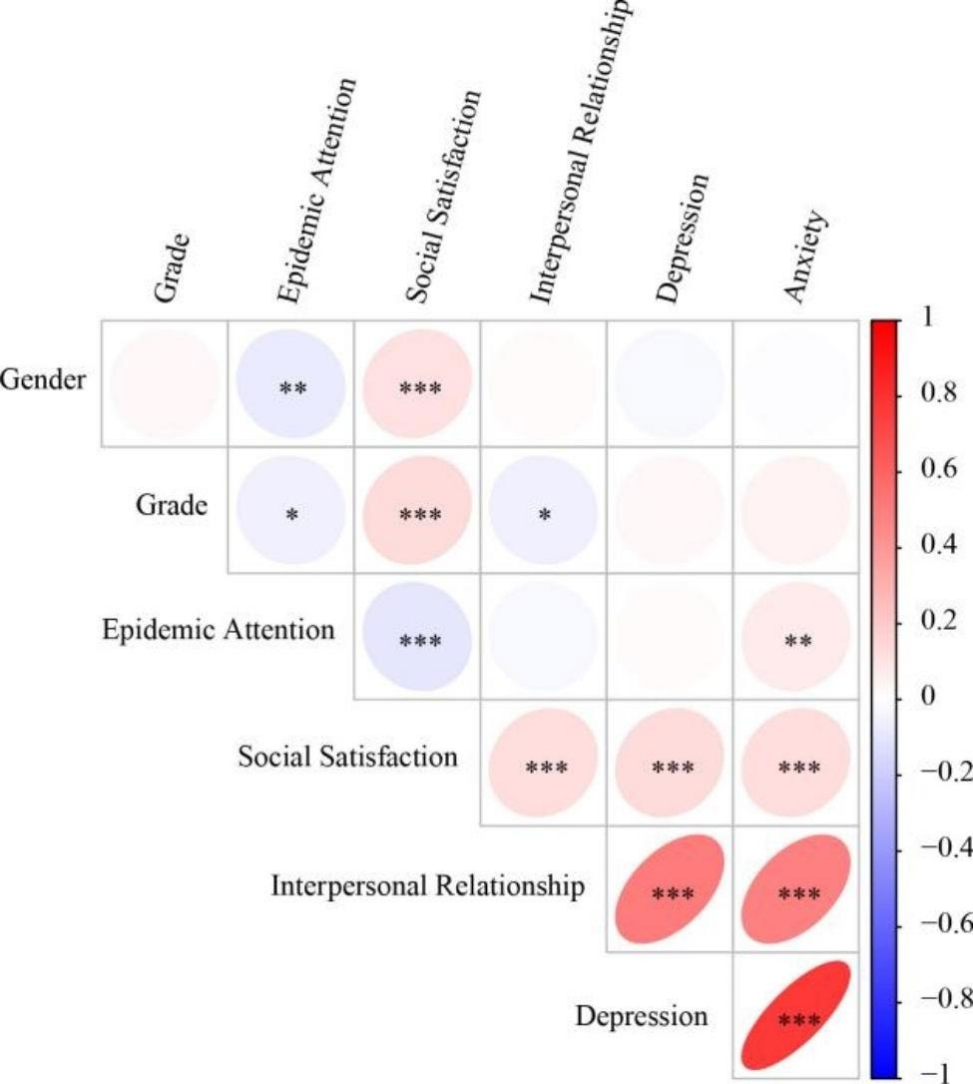
Correlation heat map

The means, standard deviations, and correlation coefficients among the study variables are presented in Table 1. Additionally, Fig. 1 displays the correlation heat map.

During the pandemic, Pearson’s correlations showed that gender was significantly negatively correlated with epidemic attention (r = -0.076, p < 0.001) but positively correlated with social satisfaction (r = 0.112, p < 0.001). Grade was significantly negatively correlated with epidemic attention (r = -0.054, p < 0.05) and relationship (r = -0.057, p < 0.05) but positively with social satisfaction (r = 0.133, p < 0.001). Epidemic attention was significantly negatively correlated with social satisfaction (r = -0.098, p < 0.001) but positively correlated with anxiety (r = 0.078, p < 0.001). Social Satisfaction was significantly positively correlated with relationship (r = 0.129, p < 0.001), depression (r = 0.140, p < 0.001) and anxiety (r = 0.120, p < 0.001). Relationship was significantly positively correlated with depression (r = 0.519, p < 0.001) and anxiety (r = 0.497, p < 0.001). There was a significant positive correlation between depression and anxiety (r = 0.77, p < 0.001).

**Table 1:** Description statistics and correlations

Variables	Mean	SD	1	2	3	4	5	6	7
1.Gender(1 = male)	1.72	0.448	1						
2.Grade	2.07	1.102	0.027	1					
3.Epidemic Attention	8.2998	2.3536	-0.076^**^	-0.054^*^	1				
4.Social Satisfaction	9.4294	3.0729	0.112^**^	0.133^**^	-0.098^**^	1			
5.Relationship	6.7726	5.9442	0.014	-0.057^*^	-0.023	0.129^**^	1		
6.Depression	33.3666	8.2287	-0.028	0.024	0.012	0.140^**^	0.519^**^	1	
7.Anxiety	33.4011	7.3278	-0.020	0.042	0.078^**^	0.120^**^	0.497^**^	0.77^**^	1

Significance level: *p < 0.05; **p < 0.001

### Model construction and variable design

#### Principles of structural equation modeling

Structural equation model is a multivariate statistical method that uses linear equations to express the causal relationship between variables based on the covariance matrix of variables. It includes two parts: the measurement model and the structural model. The matrix equation is:

1 $$X = \Lambda x\xi + \delta$$

2 $$Y = \Lambda {\rm{y}}\eta + \varepsilon$$

$$\eta = \beta \eta + \Gamma \xi + \zeta$$

(1)The formula is the measurement equation, (2) The formula is the structural equation, where X is a vector composed of exogenous indicators, and Y is a vector composed of endogenous indicators,$\xi$ is a vector of exogenous latent variables, ${\upeta }$ is a vector of endogenous latent variables$, {\Lambda }\text{x}$ is a coefficient matrix that reflects the strength of the relationship between exogenous observation variables and exogenous latent variables;${\Lambda }\text{y}$ is a coefficient matrix reflecting the strength of the relationship between endogenous observed variables and endogenous latent variables, ${\updelta }$ represents the measurement error of exogenous variables, ${\upepsilon }$ represents the measurement error of the endogenous variable, $\beta$ represents the coefficient matrix of the endogenous latent variable$,{\rm{ }}\Gamma$ represents the coefficient matrix of the exogenous latent variable, $\zeta$ represents the error of the structural equation.

#### Structural equation model and index design

**Fig. 2:**
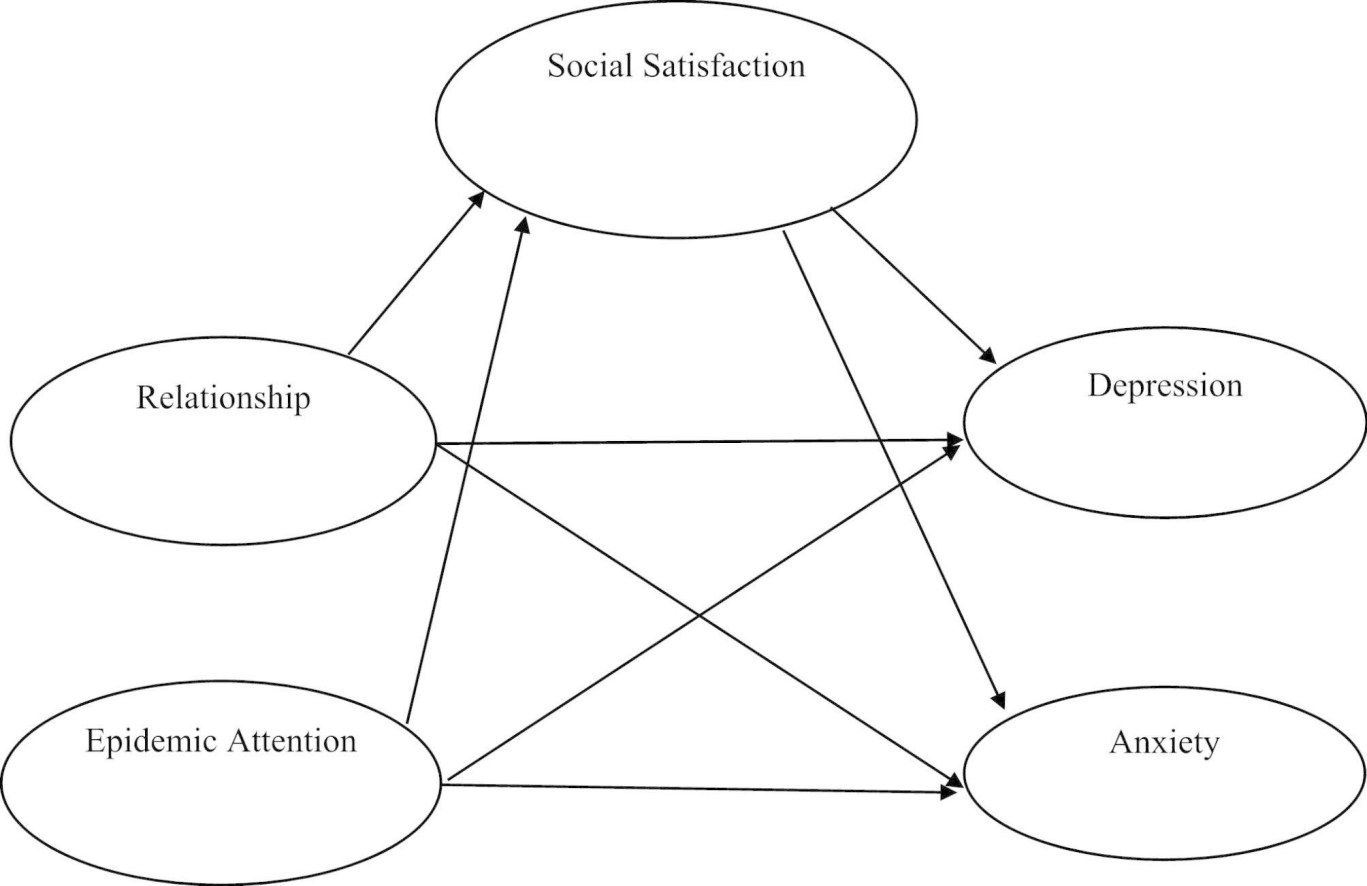
Structural relational model

This paper intends to construct a structural equation index system composed of 5 basic dimensional latent variables and 20 observation variables (Table 2). The theory posits a direct relationship between interpersonal relationships, epidemic attention, social satisfaction and anxiety and depression. To illustrate this, we have constructed a structural relationship model, as depicted in Fig. 2.

Among them, the basic dimensions of interpersonal relationships include four observed variables: conversation behavior, social networking, dealing with individuals, and heterosexual interactions. The basic dimensions of epidemic attention include the total concern about the epidemic, the time to read and view the epidemic information, the number of epidemic information obtained and the frequency of reading and viewing the epidemic information. The basic dimensions of social satisfaction include four observed variables: satisfaction with community security, satisfaction with school epidemic prevention and control measures, satisfaction with the convenience of living and satisfaction with the current state of life. The basic dimensions of depression include four observed variables: psychogenic-emotional symptoms, somatic disorders, dyskinesia and mental disorders. The basic dimensions of anxiety include four observed variables: psychogenic-emotional symptoms, somatic disorders, dyskinesia and mental disorders.

**Table 2:** The relationship between latent variables and observed variables

Latent variable	Observed variable	Code
Interpersonal relationship	Conversational behavior	x1
	Social networking	x2
	Dealing with individuals	x3
	Heterosexual interactions	x4
Epidemic attention	Total concern about the epidemic	ATT1
	Time to read and view epidemic information	ATT2
	Number of epidemic information obtained	ATT3
	Frequency of reading and watching epidemic information	ATT4
Social satisfaction	Satisfaction with community security	SAT1
	Satisfaction with school epidemic prevention and control measures	SAT2
	Satisfaction with the convenience of living	SAT3
	Satisfaction with current life status	SAT4
Depression	Psychogenic-emotional symptoms	SD1
	Somatic disorders	SD2
	Dyskinesia	SD3
	Mental disorder	SD4
Anxiety	Psychogenic-emotional symptoms	SA1
	Physical disorder	SA2
	Dyskinesia	SA3
	Mental disorder	SA4

### Reliability and validity test

The scale was tested for reliability (Table 3) and descriptive statistical analysis (Table 4) using SPSS 24.0 software, and the total scale had a Cronbach’s alpha value of 0.839, which was greater than 0.8 and had some reliability. The Cronbach’s α value of each dimension fluctuates between 0.60 and 0.86, which were all greater than 0.6, indicating that the reliability of the scale was relatively good. Besides, the KMO value and Bartlett’s sphere test were performed on the scale. The KMO value of the total scale and each dimension fluctuated between 0.654 and 0.878, all greater than 0.6, and the t-test values were also significant at the 0.05 level, indicating the structural model of performance’s validity was good.

**Table 3:** Reliability and validity test results

Category	Cronbach’s Coefficient	KMO value	Bartlett’s sphericity test		
			Approximate chi-square	Degree of freedom	Significance
Total scale	0.839	0.878	10939.836	190	***
Interpersonal relationship	0.860	0.801	2915.472	6	***
Epidemic attention	0.60	0.654	596.858	6	***
Social satisfaction	0.70	0.696	957.324	6	***
Anxiety	0.675	0.689	1317.869	6	***
Depression	0.748	0.793	2173.061	6	***

**Table 4:** Basic characteristics of observed variable data

Indicator code	Mean	Standard deviation	Factor loading
ATT1	2.980	0.731	< 0.60
ATT2	1.280	0.672	0.613
ATT3	1.650	0.886	0.777
ATT4	2.380	1.149	0.716
SAT1	2.080	0.731	0.723
SAT2	2.170	1.112	0.762
SAT3	2.710	1.093	0.706
SAT4	2.480	1.023	0.686
X1	1.8463	1.860	0.875
X2	2.4652	2.152	0.900
X3	0.8842	1.273	0.786
X4	1.5768	1.682	0.815
SD1	3.024	1.180	0.740
SD2	12.828	3.250	0.851
SD3	3.888	1.296	0.814
SD4	13.627	4.009	0.846
SA1	6.130	2.220	0.789
SA2	11.253	3.288	0.786
SA3	12.355	3.073	0.663
SA4	3.664	1.161	0.734

### Model fit test and correction

**Fig. 3:**
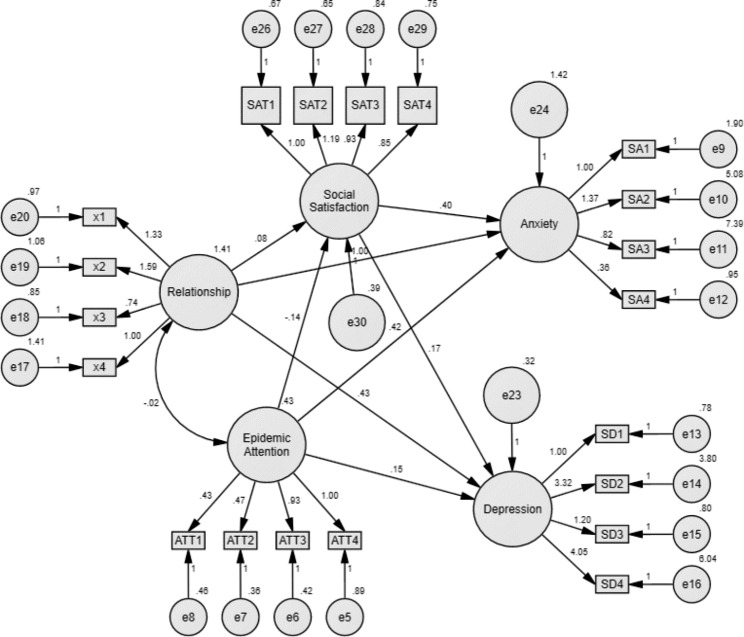
The initial structural equation model

To explore the relationship and action path of the negative emotions of college students during the epidemics, we used AMOS22.0 software to build a structural equation model and used the maximum likelihood method to estimate the initial model. The initial model was shown in Fig. 3.

**Fig. 4:**
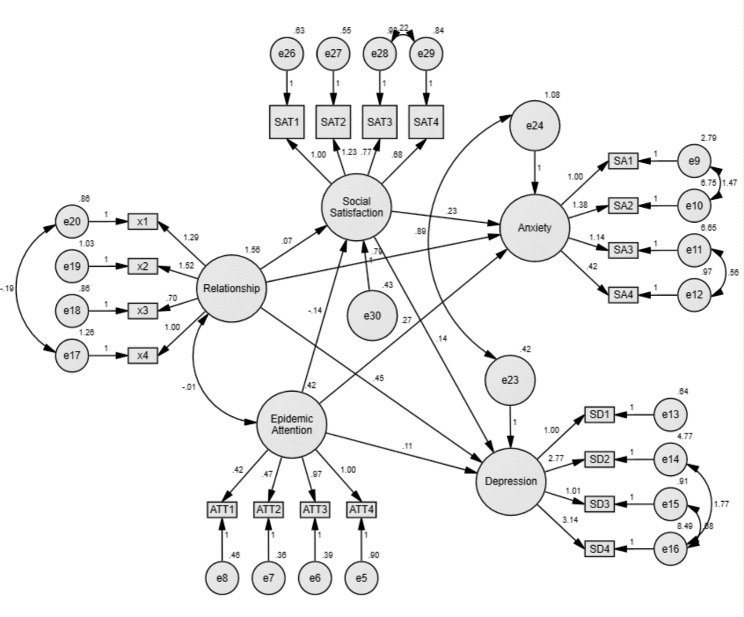
The revised structural equation model

After the initial model simulation, the results showed that the correction index MI value between the four latent variables of anxiety, depression, social satisfaction and interpersonal relationship was relatively large. The relevant path could be added to modify the model, and increased [e9-e10], [e14 -e15] equal residual path. The p-value of each path after the correction was less than 0.05, which was statistically significant. The final model was shown in Fig. 4. Due to the large sample size in this study, the CMIN/DF index was too large, but the fitting results of other fitness indexes were good. The fitting results were shown in Table 5.

**Table 5:** Model fit index

Fitting index		Judgment value	Initial model	Revised model
Absolute fit index	CMIN/DF	1 ~ 3	7.894	5.919
	GFI	≥ 0.9	0.906	0.941
	RMSEA	< 0.05	0.069	0.058
Value-added fitting index	AGFI	≥ 0.9	0.877	0.920
	NFI	≥ 0.9	0.875	0.917
	CFI	≥ 0.9	0.889	0.930
	IFI	≥ 0.9	0.889	0.930
Simplified fitting index	AIC	The smaller the better	1362.966	1023.587
	CAIC	The smaller the better	1676.966	1375.267

### Path analysis of the revised model

#### Direct effect

As shown in Table 6, interpersonal relationship disturbance had a negative effect on social satisfaction, and the standard path estimate was 0.136 (p < 0.001). Epidemic attention had a positive effect on social satisfaction. The standard path estimate was − 0.131 (p < 0.001). Interpersonal relationships and epidemic attention had a positive effect on depression and anxiety, and the standard path coefficients were 0.642, respectively. 0.676, 0.08, 0.119 (p < 0.05). Social satisfaction had a negative effect on depression and anxiety, and the standard path coefficients were 0.104 and 0.112 (p < 0.001), respectively.

**Table 6:** The effect relationship between the factors in the fitted model

Variable			Standard path estimate	C.R.	p
Social satisfaction	<--	Interpersonal relationship	0.136	4.083	***
Social satisfaction	<--	Epidemic attention	-0.131	-3.333	***
Anxiety	<--	Epidemic attention	0.119	3.290	0.001
Depression	<--	Epidemic attention	0.080	2.544	0.011
Anxiety	<--	Social satisfaction	0.104	2.986	0.003
Depression	<--	Social satisfaction	0.112	3.617	***
Anxiety	<--	Interpersonal relationship	0.676	17.904	***
Depression	<--	Interpersonal relationship	0.642	18.874	***

***means *p* < 0.001

#### Mediation effect

In this study, the Bootstrap method with deviation-corrected non-parametric percent position confidence interval was employed to test the mediation effect. The number of repeated random sampling was set to 1000, and the significance was determined by examining whether the 95% confidence interval contained 0.

From Table 7, we observe that interpersonal relationships positively influenced depression and anxiety through social satisfaction, with mediating effects of 0.015 and 0.014 (p = 0.002) respectively. Additionally, epidemic attention negatively affected depression and anxiety through social satisfaction, with mediating effects of -0.015 and − 0.014 (p ≤ 0.002).

**Table 7:** Analysis of the mediating effect between interpersonal relationships, epidemic attention, social satisfaction and negative emotions

Intermediary path	Mediation effect	95%CI		Significance
		Lower limit	Upper limit	
Interpersonal relationship → Social satisfaction → Depression	0.015	0.006	0.03	0.002
Interpersonal relationship→ Social satisfaction →Anxiety	0.014	0.005	0.031	0.002
Epidemic attention → Social satisfaction →Depression	-0.015	-0.031	-0.005	0.001
Epidemic attention → Social satisfaction →Anxiety	-0.014	-0.029	-0.004	0.002

## Discussions

This study utilized a questionnaire to explore the relationship and internal mechanism among interpersonal relationships, epidemic attention, social satisfaction and negative emotions among 1,451 students from a university in Sichuan Province. The findings revealed that social satisfaction played an intermediary role in the influence of interpersonal relationships and epidemic attention on negative emotions during the epidemic. Additionally, interpersonal relationships, epidemic attention, and social satisfaction significantly had significant effects on negative emotions. Furthermore, interpersonal relationships and epidemic attention significantly affect social satisfaction. This research contributes to a better understand of the factors influencing individuals’ negative emotions during the epidemics and sheds light on their internal connections. It also provides suggestions for regulating emotions and fostering a healthy psychological state.

### Interpersonal relationships positively predicted the level of anxiety and depression

Interpersonal relationships positively predicted the level of anxiety and depression, which was consistent with the hypothesis of this study, and also verified the results of previous studies. The more serious the interpersonal relationship is, the more likely it is to trigger and aggravate depression and anxiety. During the epidemics, the fear of pandemics among the masses led to social isolation, the face-to-face contact between individuals decreases and interpersonal relationships tend to loosen. Therefore, a negative interpersonal atmosphere was easily created, which intensified negative emotional experiences. To a certain extent, poor interpersonal relationships also represent a lack of social support, inhibiting individuals’ positive thinking about the epidemic environment, increasing the level of internal stress, and causing anxiety, depression and other emotions.

### Epidemic attention positively predicted anxiety and depression

The degree of epidemic attention positively predicted the level of anxiety and depression, which is consistent with the hypothesis of this study, and also verify the results of previous studies. Paying attention to the increase in the frequency and intensity of epidemic information is an individual’s normal objective behavior in the new risk environment [33, 34]. However, the increase in the degree of attention to the epidemic points to an increase in negative emotional experience. This was manifested in the undesirable results brought about by the behavior of overly paying attention to the epidemic information and the negative effects brought about by the negative information. Focusing on the phone for a long time is prone to the feeling of emptiness [47].

Excessive attention to information easily diminishes the ability to regulate emotions and cognitive processing. Increased focus on the epidemic also contributes to a decline in overall quality of life, resulting in a lack of recreational and sports activities, which further exacerbate negative emotions. Network information is diverse and mixed. Compared with positive information, negative information is more likely to attract the attention of the crowd. When individuals are exposed to an abundance of negative information, they become susceptible to perceiving higher levels of risk, leading to feelings of panic and anxiety. With an excessive amount of negative information received, a more intense sense of fear could be triggered [48] due to a reduced sense of control, which is at the core of anxiety generation and maintenance [49].

### Social satisfaction negatively predicted the level of anxiety and depression

Social satisfaction negatively predicted the level of anxiety and depression, which is consistent with the hypothesis of this study, that is, the higher the social satisfaction, the lower the level of anxiety and depression, and the weaker the negative emotional experience. McMillan and Chavez once proposed that when individuals’ community satisfaction is higher, the individual’s sense of attachment and belonging to the community is stronger [50]. A society is a collection of several communities. When individuals are more satisfied with society, they are more likely to have a sense of trust and security, thereby reducing the generation of negative emotions.

The university’s efforts to enhance outbreak science may be a breakthrough in reducing negative emotional experiences. Firstly, teachers reaching out more to students and building social support from teachers to students. Secondly, promoting epidemic prevention policies and knowledge as a school will not only reduce rumor generation and achieve disbelief and disinformation, but will also increase the authority and trust of the school and improve university students’ satisfaction at school, which in turn will improve social satisfaction and thus reduce the likelihood of depression and anxiety.

### Interpersonal disturbance negatively predicts social satisfaction

Interpersonal disturbance negatively predicts social satisfaction, that is, the higher the degree of interpersonal disturbance, the lower social satisfaction, which is consistent with the hypothesis of this research. The fear of pandemics among masses during the epidemic forbid unnecessary face-to-face close contact and communication between individuals such as parties which undergraduate students like very much, and individuals may be more inclined to extend the negative emotions brought about by interpersonal distancing into dissatisfaction with social policies.

### Epidemic attention positively predicts social satisfaction

The degree of epidemic attention positively predicts social satisfaction, that is, the higher the degree of epidemic attention, the higher the social satisfaction, which is consistent with the hypothesis of this study. During the epidemic, the public had higher expectations for the performance of government departments. As the main source of propagating correct epidemic information and effective epidemic prevention knowledge, the official media has become one of the directions for the public to maintain high attention. The masses maintain a high attention to the epidemic while also experiencing the quality of services provided by the government during the epidemic, to obtain A sense of security, more trust in the government [51].

In addition, the pandemic outbreak is a global public health emergency. As individuals focus on more information about the domestic outbreak, they will also receive information about other countries’ preparedness measures and their effectiveness. Government prevention and control of the outbreak has controlled the increase in domestic infections, and China has provided assistance to other countries in their prevention and control of the outbreak. These initiatives have led to an increased sense of security among university students, which in turn has led to increased social satisfaction.

### Social satisfaction partially mediates the influence of interpersonal disturbance and epidemic attention on negative emotion levels

Interpersonal problems can not only directly affect negative emotion levels but also indirectly affect negative emotion levels through social satisfaction. This shows that individuals with bad interpersonal relationships are more likely to be dissatisfied with their social environment, and are more likely to feel helpless and induce the development and escalation of anxiety and depression.

Epidemic attention indirectly predicted negative emotion levels by influencing social satisfaction, that was when individuals spend more time and attention in using correct and credible information channels to obtain real epidemic news and rationally recognize government decisions. When they understand the quality of service and other aspects, with social satisfaction increasing, the experience of depression and anxiety will decrease.

The pathognomonic features of anxiety disorders and anxiety symptoms are generally believed to be the unconscious conflicts in the psychodynamic theory [52]. On the other hand, depression symptoms are often triggered by disruption of profit and avoid loss avoidance mechanism, as well as the obstruction of the safety path and the value path [53]. Those conflicts and destruction not only come from inside, but also happens between people and within families, groups, and systems [52]. Conflicts which direct anxiety symptoms manifest themselves in the battle between inner desire and standard imposed by society or environment [52]. After perceiving the negative stimulus which is imminent to the psychological limit, individuals are prone to experience anxiety [53]. During the period of epidemics, the strong desire to go out with friends clashed with the strict social requirement to isolate oneself at home, leading to lower social satisfaction and aggravate anxiety. However, due to the control of negative epidemic information and the dissemination of positive information, it can not only enhance the public’s awareness of social management during the epidemic and increase social satisfaction, but also reduce the threat to life. This declines the conflict between survival instinct and the danger of the epidemic helps decrease the level of anxiety.

Depression begins with the breaking of psychological limits, which refers to minimum expectations in life. When limits are breached, negative experiences become intensify, and attention becomes more focused on negative information [53]. Negative events predict distress in the form of negative mood and depression symptoms [54, 55]. Therefore, it is necessary to control the dissemination of negative epidemic information. During pandemics, the general population tended to be more sensitive to epidemics. However, due to the effective information management, social satisfaction rose as the level of epidemic attention become higher, leading to a reduction in depression experiences. Whereas individuals were prone to experience depression when interpersonal disturbances worsened because of the necessity of isolation, resulting in reduced social satisfaction.

Therefore, the government should create a more accessible information platform for the public, publish immediate information, pay attention to the direction of public opinion, and be attentive to the expectations and voices of the public to increase their social satisfaction and avoid a negative emotional climate during the critical period of the epidemic.

## Limitations

However, this study also has some limitations. Firstly, it is currently a behavioral study only, which can be studied later by incorporating techniques from cognitive neuroscience, such as magnetic resonance and electroencephalography. Secondly, this paper only discusses the relationship between interpersonal relationships, epidemic concern, social satisfaction and negative emotions, and the impact of more than these dimensions in the context of the pandemics.

## Implication

This study could contribute to enriching theoretical research on depression and anxiety while also increasing social attention of negative emotions experienced by medical students. Furthermore, it can provide a practical foundation for emotional interventions during public health emergencies, and explore strategies for enhancing mental health in the post-epidemic era.

## Conclusions

This study examined the mediating role of social satisfaction in the influence of interpersonal relationships and epidemic attention on the level of negative emotions. The results showed that social satisfaction play a partially mediating role in the influence of interpersonal relationships and epidemic attention on depression and anxiety levels. In addition, the interpersonal disturbance has a negative predictive effect on social satisfaction, epidemic attention has a positive predictive effect on social satisfaction, social satisfaction negatively predict negative emotional level and interpersonal disturbance and epidemic attention are positive to predict the level of anxiety and depression. In the post-epidemic era, support from family, school and society should be enhanced to improve the social satisfaction of medical students. Negative emotions should be found immediately and targeted strategies should be formulated to improve mental health problems among medical students.

## Data Availability

The datasets in the current study of this study are available from the corresponding author on reasonable request.
